# Deciphering the substrate recognition mechanisms of the heparan sulfate 3-*O*-sulfotransferase-3†

**DOI:** 10.1039/d1cb00079a

**Published:** 2021-05-28

**Authors:** Rylee Wander, Andrea M. Kaminski, Yongmei Xu, Vijayakanth Pagadala, Juno M. Krahn, Truong Quang Pham, Jian Liu, Lars C. Pedersen

**Affiliations:** Division of Chemical Biology and Medicinal Chemistry, Eshelman School of Pharmacy, University of North Carolina Chapel Hill North Carolina USA jian_liu@unc.edu; Genome Integrity and Structural Biology Laboratory, National Institute of Environmental Health Sciences, National Institutes of Health Research Triangle Park North Carolina USA; Glycan Therapeutics Raleigh North Carolina USA

## Abstract

The sulfation at the 3-OH position of a glucosamine saccharide is a rare modification, but is critically important for the biological activities of heparan sulfate polysaccharides. Heparan sulfate 3-*O*-sulfotransferase (3-OST), the enzyme responsible for completing this modification, is present in seven different isoforms in humans. Individual isoforms display substrate selectivity to uniquely sulfated saccharide sequences present in heparan sulfate polysaccharides. Here, we report two ternary crystal structures of heparan sulfate 3-OST isoform 3 (3-OST-3) with PAP (3′-phosphoadenosine 5′-phosphate) and two octasaccharide substrates: non 6-*O*-sulfated octasaccharide (8-mer 1) and 6-*O*-sulfated octasaccharide (8-mer 3). The 8-mer 1 is a known favorable substrate for 3-OST-3, whereas the 8-mer 3 is an unfavorable one. Unlike the 8-mer 1, we discovered that the 8-mer 3 displays two binding orientations to the enzyme: productive binding and non-productive binding. Results from the enzyme activity studies demonstrate that 8-mer 3 can contribute to either substrate or product inhibition, possibly attributed to a non-productive binding mode. Our results suggest that heparan sulfate substrates interact with the 3-OST-3 enzyme in more than one orientation, which may regulate the activity of the enzyme. Our findings also suggest that different binding orientations between polysaccharides and their protein binding partners could influence biological outcomes.

## Abstract 2

Co-crystallization and biochemical analyses with structurally defined oligosaccharides show the low reactivity of HS 3-OST-3 toward 6-*O*-sulfated substrates is due to inhibition of enzyme activity by 6-*O*-sulfated oligosaccharides.

## Graphical abstract

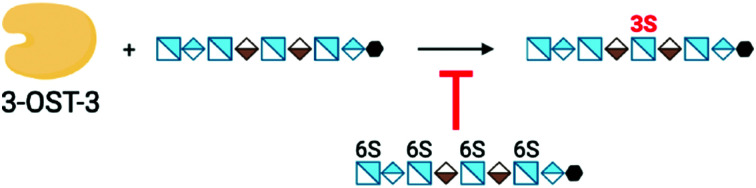

Heparan sulfate (HS) is a polysaccharide that is widely present in the human body and plays essential functions. Heparin, a special form of HS produced by mast cells, is a widely used anticoagulant drug with clinical applications for treatment of blood clotting disorders.^1^ HS consists of repeating disaccharide units of glucuronic acid (GlcA) or iduronic acid (IdoA) linked to a glucosamine (GlcN) saccharide, and each individual unit can be sulfated. Its biological function is achieved through interactions with protein effectors. Oligosaccharide length, composition, positioning of both GlcA and IdoA saccharides, and sulfation pattern of HSs play critical roles in dictating both binding affinity and specificity for protein effectors to control the biological outcomes. While heparin is primarily known for its use as a clinical anticoagulant, the biological roles of HS are much more ambiguous. HS has been implicated in a variety of biological pathways including angiogenesis, inflammation, and embryonic development but the specifics of its roles in these pathways are not known.^2–4^ Heparin is much more highly modified than HS and has greater anticoagulant activity. The potent canonical anticoagulant heparin pentasaccharide sequence known to bind antithrombin carries several modifications including *N*-sulfation, epimerization, 2-*O*-sulfation, 6-*O*-sulfation, and 3-*O*-sulfation. HS, on the other hand, carries fewer modifications and an overall decreased negative charge.^5^

The biosynthesis of HS involves a series of enzymes that regulate the structure of saccharide sequences. The backbone of heparin and HS consists of alternating units of *N*-acetylated glucosamine (GlcNAc) and glucuronic acid (GlcA) in a 1 → 4 beta linkage that is built primarily by the HS co-polymerase transferring a GlcNAc or GlcA from UDP-GlcNAc or UDP-GlcA respectively.^6^ This backbone is subsequently modified by a series of Golgi-resident enzymes including an *N*-deacetylase/*N*-sulfotransferase, various *O*-sulfotransferases, and a C_5_ epimerase.^6^ In HS, sulfation is found at the 2-OH of IdoA (and, less frequently, GlcA) and N-, 3-OH and 6-OH positions of GlcNS saccharides. The sheer number of possible permutations and extent of modifications by the sulfotransferases and *C*_5_-epimerase lead to an incredibly high level of diversity amongst HS chains, which explains the wide array of roles these molecules are known to play in the body.

There exist seven isoforms of 3-OST within the human genome, making it the largest family of HS-modifying enzymes in humans, despite the relative rarity of the 3-*O*-sulfation modification.^7^ The enzymes transfer a sulfo group to the 3-OH position of glucosamine in HS. Different isoforms of 3-OST, such as 3-OST-1 and 3-OST-3, display distinct substrate specificities that can be detected experimentally (Fig. 1A). Notably, the 3-*O*-sulfation modification has been shown to be important for bioactivities in both heparin and HS, including both the anticoagulant activity of heparin and the ability of HS to serve as an entry receptor for herpes simplex virus 1 (HSV-1).^8,9^ Different 3-OST isoforms have also been implicated in a variety of diseases including Alzheimer's disease and cancer, making the study of these enzymes an important area of research for potential therapeutic intervention.^10–13^ The isoforms of 3-OST differ in several respects, including tissue expression and substrate specificity.^7,14^ To date, only the substrate specificities of isoforms 1, 3, and 5 have been studied in detail^15–17^ and the structural mechanisms used by different 3-OST isoforms for substrate recognition of distinct saccharide sequences is not fully understood.

**Fig. 1:**
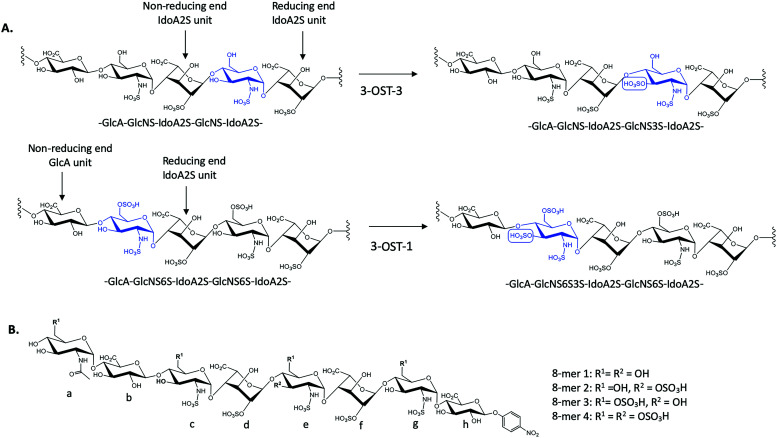
Sulfation reactions catalyzed by 3-OST-1 and 3-OST-3 and chemical structures of four octasaccharide substrates used for the study. (A) Sulfation reactions by 3-OST-3 (top) and 3-OST-1 (bottom). The acceptor saccharides are colored blue and the flanking reducing and non-reducing saccharides of the acceptor sites are indicated. The position of 3-*O*-sulfation is circled for clarity. (B) Chemical structures of four octasaccharides used for this study. All four 8-mers were synthesized *via* the chemoenzymatic approach.

3-*O*-Sulfation is reportedly the last modification step in the biosynthetic pathway of HS, occurring after 6-*O*-sulfation.^18^ A recent study by Wang *et al* suggested 3-*O*-sulfation can occur before the 6-*O*-sulfation step, depending on the 3-*O*-sulfotransferase isoform.^16^ The 3-OST-1 isoform utilizes a substrate with a GlcA on the non-reducing side, adjacent to the GlcNS acceptor, while the 3-OST-3 isoform utilizes a substrate with an IdoA2S at this position (Fig. 1A). Using homogeneous oligosaccharide substrates, the previous study demonstrated that 3-OST-3 was very active towards substrates lacking 6-*O*-sulfation, while 3-OST-1 was practically inactive.^16^ To better understand the molecular underpinnings of substrate specificity differences between these two enzymes, we solved the crystal structures of 3-OST-3 complexed with two distinct octasaccharide substrates, 8-mer 1 (non-6-O-sulfated octasaccharide) and 8-mer 3 (6-*O*-sulfated octasaccharide) (Fig. 1B). Biochemical and mutagenesis studies were conducted to investigate the substrate specificity. No specific structural motif in 3-OST-3 is solely accountable for excluding saccharide substrates containing 6-*O*-sulfation. However, we discovered that 6-*O*-sulfated oligosaccharide substrates, *i.e.* 8-mer 3, exhibited substrate and product inhibition to the enzyme activity, possibly stemming from a non-productive enzyme/substrate interaction. Our findings suggest that highly sulfated HS may cause different binding orientations between HS and proteins, resulting in different biological outcomes.

## Results

To characterize the structure and function of 3-OST-3, we synthesized four different octasaccharides (8-mer 1 to 8-mer 4) (Fig. 1B) using the chemoenzymatic approach.^16,19^ 8-mer 1 and 8-mer 3, octasaccharides without or with 6-*O*-sulfation respectively, are presumably the substrates for the 3-OST-3 enzyme. The two remaining octasaccharides, 8-mer 2 and 8-mer 4, carry a 3-*O*-sulfo group on saccharide *e* (Fig. 1B), and therefore represent the product forms of 8-mer 1 and 8-mer 3, respectively, after 3-OST-3 sulfation. All four octasaccharides were synthesized at 10–100 mg scale with purity >93%, as determined by high resolution anion exchange HPLC (Fig. S1–S4, ESI†). The molecular weight of each compound was determined by ESI-MS, confirming the structure (Fig. S1–S4, ESI†).

### Comparison of the reactivity and binding affinity of 8-mer 1 and 8-mer 3 to 3-OST-3 enzyme

**Fig. 2:**
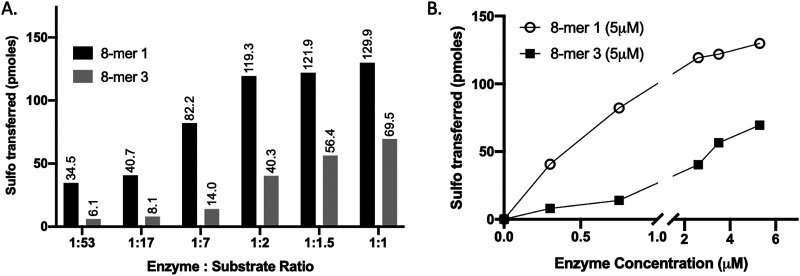
Increasing enzyme concentration relative to substrate alters the selectivity of 3-OST-3, enabling sulfation of a 6-*O*-sulfated substrate (8-mer 3). (A) Titration of 3-OST-3 enzyme concentration using either 8-mer 1 (black bar) or 8-mer 3 (grey bar) at constant substrate concentration. Both reactions show increased progression, measured as pmoles of sulfo groups transferred, with increasing enzyme to substrate ratio. This suggests both oligosaccharides are viable substrates for 3-OST-3. (B) Graph depicting the trend in sulfo groups transferred with increasing enzyme concentration for both 8-mer 1 (open circle) and 8-mer 3 (closed square). As enzyme concentration increases, the difference between the 8-mer 1 and 8-mer 3 lines decreases, suggesting the selectivity of 3-OST-3 toward non-6-*O*-sulfated substrates is enzyme-concentration dependent.

We compared the susceptibility of 8-mer 1 and 8-mer 3 to 3-OST-3 modification based on the amount of ^35^S-labeled products after the reaction (Fig. 2). Previously, it was reported that 3-OST-3 does not effectively sulfate oligosaccharides carrying 6-*O*-sulfation.^16^ As expected, the results revealed that, at lower molar ratios of enzyme to substrates, 3-OST-3 sulfated 8-mer 1 ∼6-fold more than 8-mer 3. As the molar ratio of enzyme to substrate increased to 1 : 1, the difference in ^35^S-labeled products from the two substrates, however, decreased to ∼2-fold (Fig. 2A and B). This suggests that 6-*O*-sulfated oligosaccharides, like 8-mer 3, can be substrates for 3-OST-3, but with lower reactivity.

**Fig. 3:**
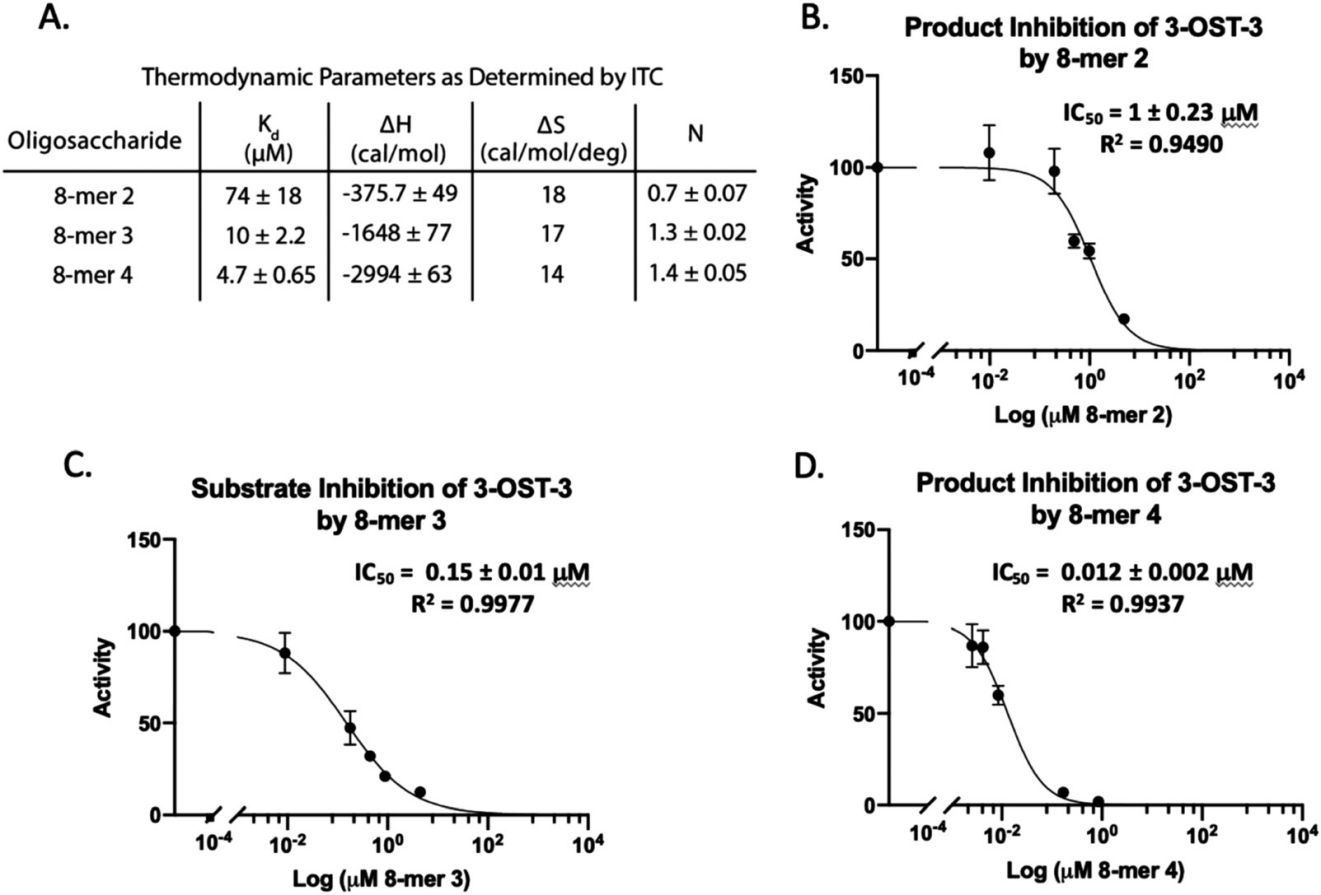
Product and substrate inhibition of 3-OST-3. (A) Isothermal titration calorimetry data obtained for the binding interaction of 3-OST-3 with 8-mers 2, 3, and 4. The product of 8-mer 1 sulfation, 8-mer 2, shows relatively weak product inhibition (B), while the 6-sulfated 8-mer 3 substrate displays increased inhibition (C). 3-*O* Sulfation of 8-mer 3 (8-mer 4) is the most potent inhibitor of the 8-mers tested (D). The increased binding affinity of the more sulfated octasaccharides likely contributes to increased potency of inhibition.

Isothermal titration calorimetry (ITC) was then used to determine the binding affinity of each octasaccharide to 3-OST-3. We discovered that addition of sulfo groups on an octasaccharide increases the binding affinity to 3-OST-3 (Fig. 3A). ITC analysis was unable to detect binding of the 8-mer 1 substrate to 3-OST-3, suggesting a low binding affinity. The 8-mer 2, a product of 8-mer 1 after 3-OST-3 modification, displayed a *K*_d_ value of 74 μM. Addition of 6-*O*-sulfo groups to the octasaccharide (8-mer 3) yielded a *K*_d_ value of 10 μM, and the addition of 3-*O*-sulfation to the octasaccharide 8-mer 3, 8-mer 4, further decreased the *K*_d_ value to 4.7 μM. These results suggest that increased sulfation on these oligosaccharides increases binding affinity to 3-OST-3. Furthermore, the ITC analysis indicates that the poor reactivity of 3-OST-3 towards 8-mer 3 cannot be solely attributed to low binding affinity, since binding of the preferred substrate, 8-mer 1, was undetectable in the assay. Higher binding affinity of 8-mer 3 to 3-OST-3, combined with low reactivity, suggests that increases in 6-*O*-sulfo groups in the substrate may contribute to inhibition.

### 8-mer-3 displays both substrate and product inhibition to 3-OST-3

Inhibition studies were performed to investigate if 6-*O*- and 3-*O*-sulfated oligosaccharides could contribute to inhibition of 3-OST-3 (Fig. 3B–D). The 8-mer 2 displayed relatively weak inhibition with an IC_50_ of around 1 μM (Fig. 3B) suggesting little product inhibition against 8-mer 1 sulfation. The 8-mer 3 has an IC_50_ value of 0.15 μM (Fig. 3C), suggesting that addition of 6-*O*-sulfation increases 8-mer 3 substrate inhibition to the enzyme. The 8-mer 4, an octasaccharide that contains both 3-*O*- and 6-*O*-sulfation, had an IC_50_ value of 0.012 μM, displaying even stronger inhibition of 3-OST-3 than 8-mer 3 (Fig. 3D). The inhibition of 3-OST-3 by 8-mer 4 is indicative of product inhibition from 8-mer 3, due to the fact it is a product from 8-mer 3 after 3-OST-3 modification.

**Fig. 4:**
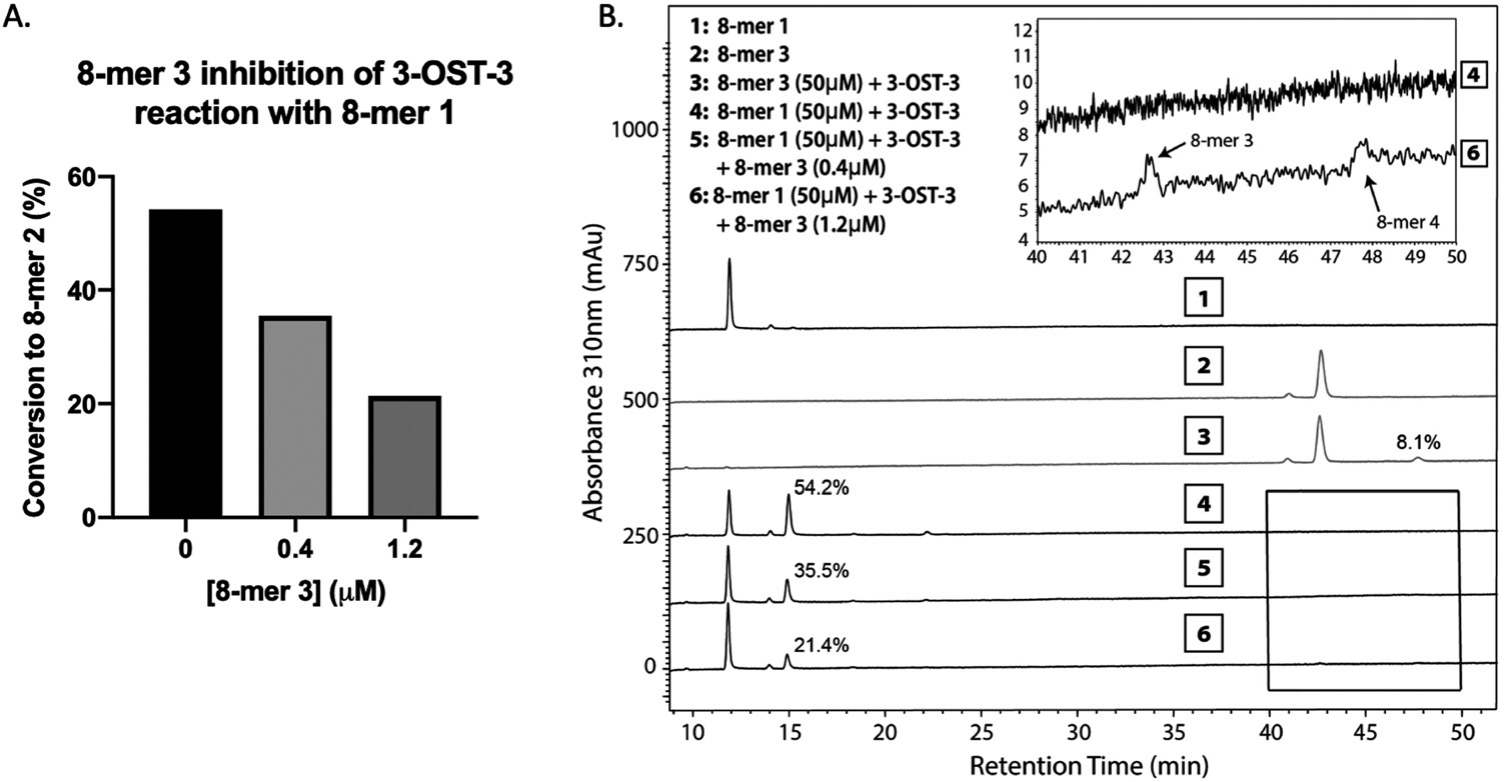
Product analysis of 3-OST-3 modified 8-mer 1 in the presence or absence of 8-mer 3. (A) Conversion of 8-mer 1 to 8-mer 2 in the presence of different concentrations of 8-mer 3. The conversion percentage was calculated based on the relative peak areas of 8-mer 1 and 8-mer 2 after HPLC analysis. (B) HPLC chromatography analysis of six reaction mixtures. The designation for each reaction mixture is indicated. Inset shows the enlarged regions where 8-mer 3 and 8-mer 4 elute, displaying some conversion of 8-mer 3 to 8-mer 4.

Next, we confirmed the inhibition effect from 8-mer 3 using a HPLC-based, non-radioactive assay. Here, the 8-mer 1 (50 μM) was incubated with 3-OST-3 enzyme in the absence or presence of different concentrations of 8-mer 3. The reaction products were then resolved by anion exchange HPLC. The analysis allowed us to determine the extent of conversion of 8-mer 1 to 8-mer 2 as well as the conversion of 8-mer 3 to 8-mer 4 (Fig. 4). As expected, 3-OST-3 exhibited reduced formation of 8-mer 2 from 54.2% to 35.5% in the presence of 0.4 μM 8-mer 3, and was further reduced to 21.4% in the presence of 1.2 μM 8-mer 3 (Fig. 4A). It should be noted that 59% of 8-mer 3 was converted to 8-mer 4 when 8-mer 3 was employed in the reaction mixture (Fig. 4B, inset). This result suggests that the inhibition effect observed was due to both 8-mer 3 and 8-mer 4 being present. Taken together, our data suggest that the inhibition of 8-mer 1 sulfation by 8-mer 3 is a function of both substrate and product inhibition.

### Binding of 8-mer 1 to 3-OST-3

**Fig. 5:**
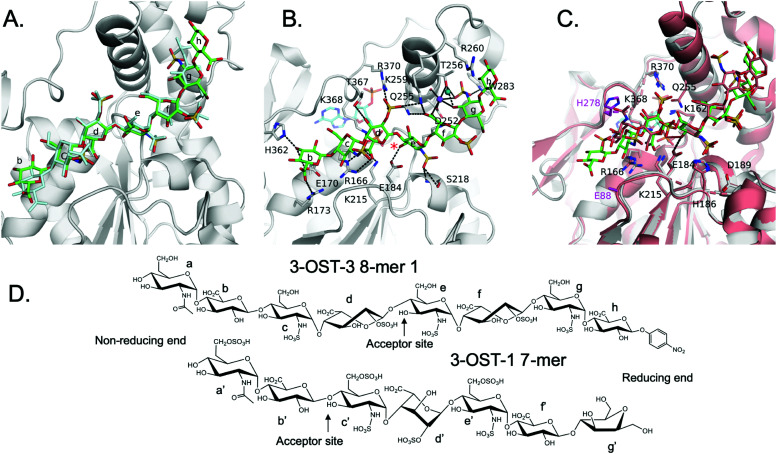
Structure analysis of 8-mer 1 Binding to 3-OST-3 (A) Position of the two 8-mer 1 oligosaccharides in the active site of 3-OST-3 based on the superposition of the two 3-OST-3 molecules (molecule B of 3-OST-3 shown, RMSD of 0.40 Å over 257 Cα atoms). 8-mer-1 (green) from active site of molecule B (gray). The 8-mer 1 from molecule A is colored all in light blue. Saccharides are labeled as in Fig. 1B. (B) Binding interactions of 3-OST-3 molecule B with 8-mer 1 substrate (green) and PAP cofactor product (cyan). Potential hydrogen bonds are shown in black dashed lines, and the bound sodium ion is colored purple with a nearby iodide ion from the crystallization condition colored aqua. A red asterisk denotes the position of the acceptor 3-OH for the sulfo transfer. (C) Superposition of the structure of 3-OST-1 (pink, PDB ID code 3UAN^21^ molecule A) onto 3-OST-3 molecule B bound to 8-mer 1 (RMSD of 0.87 Å over 248 Cα atoms). Conserved residues in 3-OST-1 and -3 previously shown to be important for activity (Lys162, Arg166, Glu184, His186, Asp189, Lys215, Gln255, Lys368, Arg370) are displayed for both enzymes. Shown in magenta are the two gate-residues of 3-OST-1, His278 and Glu88 (sidechain is disordered and not modeled), that are important for 3-OST-1 specificity.^17^ The hydrogen bond between the catalytic base Glu184 and acceptor 3-OH is represented with a dashed line, while the trajectory of the in-line transfer from the acceptor 3OH to the leaving group PAP is highlighted with a red dashed line. (D) Chemical structures of the ordered saccharides from the 8-mer 1 substrate bound to 3-OST-3 (top) and the chemical structure of the visible saccharides from the 7-mer bound in the 3-OST-1 structure (3UAN^21^) (bottom).

We solved the crystal structures of 3-OST-3 in the presence of PAP, the sulfo donor product analog, and 8-mer 1 at 2.34 Å, revealing how the enzyme interacts with the octasaccharide (Table 1 and Fig. S1A, ESI†). The crystal structure of 8-mer 1 contains two molecules of 3-OST-3 in the asymmetric unit, each with bound PAP and 8-mer 1 in the active site. Superposition of the two 3-OST-3 molecules (RMSD 0.40 Å over 257 Cα atoms) reveals that the two 8-mer 1 substrates are observed in similar orientations binding along an open binding cleft (Fig. 5A). The substrates exhibit strongest similarity at the acceptor GlcNS (saccharide *e*) and show minor differences at both the non-reducing and reducing ends (Fig. 5A). The active site in molecule B has better quality electron density for the octasaccharide and sidechains (Fig. S5A, ESI†) and was therefore used for discussion in this study. The crystal structure reveals a significant number of interactions between saccharides *b-g* and active site residues (Fig. 5B and Table S1, ESI†). A crystal structure of 3-OST-3 binding to a tetrasaccharide, which has a Δ_4,5_ unsaturated 2-*O*-sulfo iduronic acid at the nonreducing end, was previously reported.^20^ The structure provided information on the position of the acceptor GlcNS and its adjacent saccharides but does not address the extended binding cleft interactions or explain why 3-OST-3 has diminished activity towards 6-*O*-sulfated oligosaccharide substrates.^20^ The general position of the 8-mer 1 is similar to that obtained in the crystal structure of 3-OST-1 with a heptasaccharide (Fig. 5C).^21^ The location and directionality of substrate binding within this cleft is similar to that observed in 2-*O*-sulfotransferase and 3-OST-1,^21,22^ but differs from that seen in the 6-*O*-sulfotransferase.^23^ In the superposition of 3-OST-1 and 3-OST-3, both the catalytic glutamates of 3-OST-1 (Glu90) and 3-OST-3 (Glu184) and the acceptor 3-OH from the two substrates superimpose well, with the 3-OH groups located 5.6 Å and 6.0 Å from the PAP 5-phosphorous atoms, respectively. These distances are consistent with a productive binding mode for an in-line transfer reaction mechanism (Fig. 5C).^20,21^ Structural differences exist in the uronic acid conformations flanking the acceptor GlcN (Fig. 5). For 3-OST-1, the IdoA2S on the reducing side of the acceptor glucosamine is found in the ^1^*C*_4_ chair conformation (saccharide *d*′, Fig. 5D, bottom) while in 3-OST-3 it exists in a ^2^*S*_0_ skew boat conformation (saccharide *f*, Fig. 5D, top). The IdoA2S on the non-reducing side of the acceptor glucosamine in the 3-OST-3/PAP/8-mer 1 complex is also in the ^2^*S*_0_ conformation (saccharide *d* in Fig. 5D), compared to the ^4^*C*_1_ GlcA in the 3-OST-1 substrate (saccharide *b*′, Fig. 5D). For 3-OST-3, the ^2^*S*_0_ conformation of saccharide d allows the 2-*O*-sulfo group to form interactions with Lys259 and Arg370, while the carboxylate group is positioned to form a bidentate interaction with Arg166 and potentially interact with Lys215 and Lys368 (Fig. 5B). A unique feature of 3-OST-3 appears to be the involvement of a sodium ion in binding saccharides *f* and *g*, which was previously seen in the binding with the tetrasaccharide substrate.^20^

**Table 1:** Crystallographic data statistics

Crystallographic data statistics		
Data set	3-OST-3/PAP/8-mer 1	3-OST-3/PAP/8-mer 3
Space group	*P*2_1_	*C*2
Unit cell	*a* = 38.22 Å, *b* = 147.47 Å, *c* = 51.04 Å; *β* = 94.35°	*A* = 133.76 Å, *b* = 65.01 Å, *c* = 92.24 Å; *β* = 124.74°
Resolution (Å)	2.34	1.55
# Of observations	70 569	229 425
Unique reflections	22 494	91 530
*R*_sym_(%) (last shell)^a^	13.6 (40.5)	5.1 (79.2)
*I*/*σI* (last shell)	7.3 (2.1)	10.9 (2.3)
Mosaicity range	1.4–1.9	0.54–0.65
completeness (%) (last shell)	94.8 (77.2)	97.0 (90.2)
Refinement statistics		
*R*_cryst_(%)^b^	21.6	16.4
*R*_free_(%)^c^	27.1	18.3
# Of waters	71	692
Overall mean B (Å)		
Protein	40.4	23.3
PAP	34.8	15.7
8-mer	48.0	28.9
Water	47.4	33.8
r.m.s. deviation from ideal values		
Bond length (Å)	0.011	0.010
Bond angle (°)	0.939	1.070
Dihedral angle (°)	14.21	14.94
Ramachandran statistics^d^		
Favored (>98%)	97.46	97.49
Allowed (>99.8%)	100.00	99.81

a *R*_sym_ = ∑(|*I*_*i*_ − 〈*I*〉|)/∑(*I*_*i*_) where *I*_*i*_ is the intensity of the *i*th observation and 〈*I*〉 is the mean intensity of the reflection.

b *R*_cryst_ = ∑||*F*_o_| − |*F*_c_||/∑|*F*_o_| calculated from working data set.

c *R*_free_ was calculated from 5% of data randomly chosen not to be included in refinement.

d Ramachandran results were determined by MolProbity.

### 8-mer 3 Binding to 3-OST-3

**Fig. 6:**
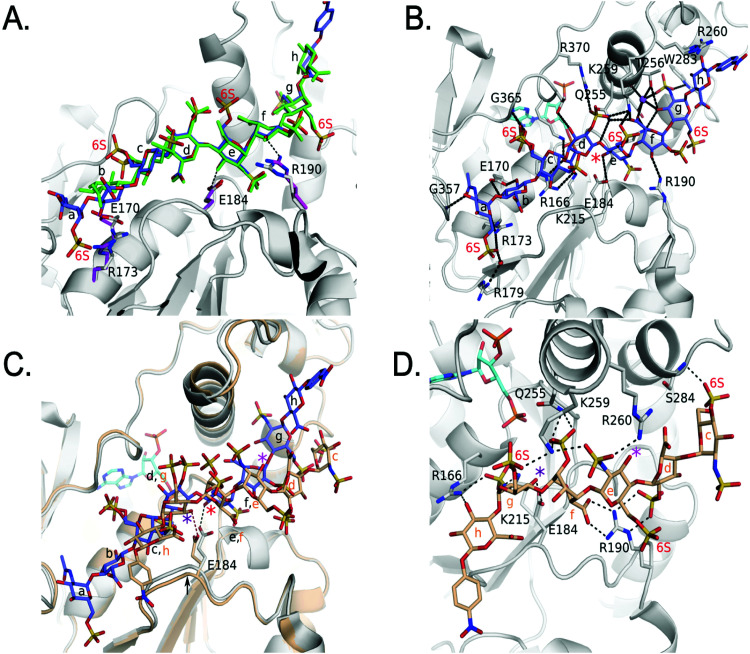
Structure analysis of 8-mer 3 Binding to 3-OST-3 (A) Superposition of 3-OST-3 (white) with 8-mer 3 (blue) bound and 3-OST-3 (not shown) with 8-mer 1 (green) bound (RMSD 0.55 Å over 256 Cα atoms). Both are in the productive binding mode. Certain residues for 3-OST-1 are shown in white and residues for 3-OST-3 are in magenta. Hydrogen bonds are shown as black dashed lines. (B) Interactions between 8-mer 3 (blue) and molecule B of 3-OST-3 (white). The acceptor 3-OH is marked with a red asterisk, sodium ion is purple and the water bridging Arg179 and the 8-mer 3 is a red sphere. (C) Superposition of 3-OST-3 molecule A (orange) and 8-mer 3 (grey) bound in non-productive mode onto 3-OST-3 molecule B with 8-mer 3 bound in the productive binding mode (8-mer 3), blue and PAP in cyan (RMSD 0.48 Å over 252 Cα atoms). The hydrogen bond between the catalytic base Glu184 and the nearest 3-OH in both structures is shown (red dashed line for molecule A and black dashed line for molecule B). The acceptor 3-OH of GlcNS6S (saccharide e) for the productive binding mode is marked with a red asterisk. The equivalent 3-OH on the non-productive binding 8-mer-3 is marked with a pink asterisk, while the 3-OH of GlcNS6S (saccharide g) near Glu184 is marked with a purple asterisk. (D) Specific interactions of 8-mer 3 binding in the non-productive mode as shown in Figure (C). The chemical structure of 8-mer 3 with differences in the ring conformation (^2^*S*_0_ productive *vs.*^1^*C*_4_ non-productive) at saccharide *f* displayed in Fig. S6 (ESI†).

We also obtained the crystal structure of 3-OST-3 bound to PAP and 8-mer 3 at 1.55 Å (Table 1 and Fig. S5B, C, Table S2, ESI†). The complex was crystallized in a different space group but with a similar asymmetric unit dimer of 3-OST-3 containing PAP and an 8-mer 3 substrate. This provides a unique opportunity to compare the interactions between 3-OST-3 with oligosaccharide substrates with or without 6-*O*-sulfation. In molecule B of the asymmetric unit, electron density exists for all saccharides and the pNP of the 8-mer 3 (Fig. S5B, ESI†). Despite the presence of 6-*O*-sulfo groups, this substrate superimposes well with the 8-mer 1 substrate, positioning the acceptor in the same position with respect to PAP (Fig. 6A). The sodium ion found on the reducing side of 8-mer 3 is also present (Fig. 6B). Three of the four 6-*O*-sulfo groups on 8-mer 3 are in position to interact with the enzyme (Fig. 6B and Table S2, ESI†). The 6-*O*-sulfo group on GlcNS6S (saccharide *a*) is within hydrogen bonding distance to Arg173 while the backbone nitrogen of Gly365 is in position to hydrogen bond with either of the two conformations of the 6-*O*-sulfo group present on GlcNS6S (saccharide *c*). The 6-*O*-sulfo group on the acceptor GlcNS6S (saccharide *e*) is in position to interact with Lys259. Notably, the presence of the 6-*O*-sulfo groups generate additional potential hydrogen interactions between 3-OST-3 and saccharides *a*, *c*, and *e* (Table S2, ESI†). These additional interactions may account for the apparent increase in binding affinity of 8-mer 3 *versus* 8-mer 1 (Fig. 3A). The presence of the 6-*O*-sulfo groups induces no significant changes in octasaccharide positioning or conformation that could explain the differences in reactivity between these two substrates (Fig. 6A, B and Table S2, ESI†). The results from the crystal structural analysis suggest that 8-mer 3 and 3-OST-3 interact productively, which should allow 8-mer 3 to serve as a substrate for the enzyme.

Surprisingly, the 8-mer 3 binds to the other molecule in the asymmetric unit in a completely different orientation (Fig. 6C, D and Fig. S5C, ESI†). A superposition of the two 3-OST-3 molecules within the asymmetric unit (RMSD 0.48 Å on 252 Cα atoms) reveals the other 8-mer 3 is binding across the cleft in the opposite direction from that of the previous oligosaccharide (Fig. 6C). The 8-mer 3 is offset such that the uronic acids, GlcA (saccharide *h*) and IdoA2S (saccharide *f*), now lie in the GlcNS6S saccharide *c* and *e* sites, and the acceptor GlcNS6S (saccharide *e*, Fig. 6E) is positioned with its 3-OH distal from the active site (Fig. 6C). Interestingly, saccharide *g* is positioned with the 3-OH within hydrogen bonding position of the catalytic base (Glu184) (Fig. 6D). However, it is located 7.4 Å away from the phosphorus atom of the 5′ phosphate and not in-line. This binding orientation is likely inconsistent with transfer of the sulfo group to the acceptor site, thereby, it is deemed to represent non-productive binding. The binding of the 8-mer 3 in this orientation also results in a shift of the loop containing Glu184 by approximately 0.8 Å away from the active site (Fig. 6C). The 6-*O*-sulfo groups form two interactions with the protein. The 6-*O*-sulfo group of saccharide *g* is located in a similar position as the 2-*O*-sulfo group from IdoA2S (saccharide *d*) in the productive binding mode, forming an interaction with Lys259 (Fig. 6B and D). In addition, the 6-*O*-sulfo group from saccharide *c* can hydrogen bond with the backbone amide of Ser284. Residues Arg166, Lys215, Gln255, Lys259, Glu184 and Arg190 all form different interactions with the 8-mer 3 non-productive binding mode.

The non-productive binding resulted in distorted interactions between enzyme and the substrate. A kink in 8-mer 3 is visible due to IdoA2S (saccharide *f*) being in the ^1^*C*_4_ conformation, as opposed to the ^2^*S*_*O*_ observed in the productive orientation (Fig. 6C, D and Fig. S5B, C, ESI†). This kink causes the non-reducing end sugars of 8-mer 3 to deviate from the canonical cleft and form extensive interactions with Arg190. This residue forms two bidentate interactions with the carboxylate of saccharide f and the 2-*O*-sulfo group from saccharide *d*, while it only forms a single interaction with the 3-OH of saccharide *f* in the productive binding mode (Fig. 6B and D). Residue Arg260, which does not interact with either 8-mer 1 or 3 in the productive binding mode, now lies within hydrogen bonding distance of the *N*-sulfo group of saccharide *e*. This structure represents the first example of a sulfotransferase binding an oligosaccharide in a non-productive binding mode. It is possible that high sulfation in the substrate increases the likelihood of binding in non-productive orientations, resulting in reduced reactivity for modification by 3-OST-3.

### Structure guided mutagenesis

Using the crystal structures as a guide, we performed a mutagenesis study focusing on residues along the cleft that might contribute to the unique specificity and properties of 3-OST-3. A previous mutagenesis study on 3-OST-3 identified residues thought to be critical for catalysis and substrate binding.^20^ The mutations K162A, R166E, E184Q, H186F, D189N, K215A, K368A, R370E of highly conserved residues and less conserved K161A and Q255A (Fig. S7, ESI†) showed less than 1% activity on a heterogeneous substrate of heparan sulfate polysaccharide and therefore were not investigated here. In this study, we examined residues along the cleft that had reasonable activity in the previous study, lacked conservation with 3-OST-1 and/or display different interactions with the substrates in the productive *versus* non-productive binding orientations or are involved in 6-*O*-sulfo binding in either binding mode (Tables S2 and S3, ESI†). In addition, Arg179 was included in this analysis since it forms a water-mediated hydrogen bond with the non-reducing end of 8-mer 3 in the productive binding mode (Fig. 6B). Based on the analysis, all of the mutants displayed a greater decrease in activity for the 8-mer-3 substrate over the preferred substrate 8-mer 1, although for the majority, the difference was slight (Table 2). However, mutations to Arg173 resulted in a loss of greater than 50% for both substrates. Arg173 is located on the non-reducing end of the binding cleft and is nestled between two glutamates (Glu170 and Glu356). Both Arg173 and Glu170 display different conformations of their sidechains to accommodate the different conformations in this region of 8-mer 1 *vs.* 8-mer 3 (Fig. 6A). The ability to form interactions with different substrates combined with maintaining proper charge balance in the non-reducing end of the pocket may explain the loss of activity of the R173A mutant.

**Table 2:** Summary of 3-OST-3 mutations made corresponding residues in 3-OST-1 and measured enzymatic activity towards 8-mer 1 and 8-mer 3 substrates

3-OST-3 Mutation	Equivalent residues in 3-OST-1	8-mer 1	8-mer 3
Wild type		100%^a^	100%^b^
R173A	S	45.7%	17.2%
R173S	S	34.1%	16.3%
R179A	A	69%	51.8%
R179E	A	98%	86.7%
R190E	W	46.8%	10%
R190A	W	64.2%	1.9%
R190K	W	94.5%	85.9%
R260E	H	104.6%	34.6%
R260A	H	148.2%	56.4%
K259A	N	29.9%	3.6%
R190E/R260E	W/H	6.2%	4.7%
S284D	K	65.6%	19.1%
S284E	K	61.8%	22.6%

a The 100% activity of 3-OST-3 towards 8-mer 1 was determined to be the transfer of 50 pmoles of sulfo groups per μg of protein in 1 hour.

b The 100% activity of 3-OST-3 towards 8-mer 3 was determined to be the transfer of 8 pmoles of sulfo groups per μg of protein in 1 hour.

Two of the mutants showed a particularly large disparity in activity towards the two substrates. Both R190A and K259A showed minimal activity for the 8-mer 3 substrate (1.9 and 3.6%, respectively), while they displayed much better activity towards 8-mer 1 substrate (64.2% and 29.9%, respectively). In the 8-mer 3 productive binding mode, Arg190 is in a slightly different orientation than when 8-mer 1 is bound, resulting in a hydrogen bond with the O3 atom of IdoA2S (saccharide *f*) and places the guanidinium group in closer proximity (3.8 Å *vs.* 4.2 Å) to the 2-*O*-sulfo group (Fig. 6A). The closer proximity to IdoA2S (saccharide *f*) may contribute to the greater loss in activity of R190A for 8-mer 3 *versus* 8-mer 1. The R190K mutant enzyme displayed similar reactivities to 8-mer 1 and 8-mer 3 as the wild type. Residue Lys259 is located at the active site and is in position to form hydrogen bonds with the carboxylate from IdoA2S (saccharide *f*) and the 2-*O*-sulfo group of IdoA2S (saccharide *d*) in both the 8-mer 1 and 8-mer 3 structures (Tables S1, S2, ESI† and Fig. 5B, 6B, respectively), but is also within hydrogen bonding distance to the 6-*O*-sulfo group of the acceptor GlcNS6S (saccharide *e*) for 8-mer 3 (Fig. 6B). For 8-mer 3, the 6-*O*-sulfo group is located merely 3.4 Å from the 2-*O*-sulfo group of IdoA2S (saccharide *d*). The positively charged amine of Lys259 may be important for reducing charge repulsion of the carboxylate and the 2-*O*-sulfo from IdoA2S (saccharide *d*), which could be particularly critical when an additional 6-*O*-sulfo group is on the adjacent saccharide.

In the non-productive binding mode of 8-mer 3, Arg190 forms bidentate hydrogen interactions with both the carboxylate of IdoA2S (saccharide *f*) and the 2-*O*-sulfo group of IdoA2S (saccharide *d*) (Fig. 6D). As well, it is only in the non-productive binding mode that Arg260 forms an interaction with 8-mer 3. It was hypothesized that mutations in these residues might reduce non-productive binding, therefore increasing the activity with the 8-mer 3 substrate. However, this turned out not to be the case. Unexpectantly, mutations in Arg260 resulted in increased activity on the 8-mer 1 substrate for R260A and reduced activity for 8-mer 3. Mutations in Arg190 had an even greater reduction in activity for 8-mer 3 than Arg260, possibly due to importance of its interactions with IdoA2S (saccharide *f*) in the productive binding orientation (Fig. 6A).

## Discussion

The 3-OST isoforms are known to introduce key modifications to confer HS polysaccharides with different biological properties, including the anticoagulant activity from HS modified by 3-OST-1 and HS modified by 3-OST-3 to serve as a viral entry receptor for herpes simplex virus 1. These iso-enzymes utilize a catalytic cleft and a series of conserved residues to bind substrate and transfer a sulfo group to the 3-OH position of a glucosamine embedded in seemingly similar polysaccharide substrates but with different saccharide sequences, conformations and sulfation patterns. Previously, we discovered a “gate structure” in 3-OST-1 to suggest a mechanism for differences in substrate specificity for 3-OST-1; but these findings do not fully explain the substrate selectivity for 3-OST-3.^16,17,21^

Here, we investigated factors contributing to the subtleties in recognizing different saccharide substrates by 3-OST-3. The ternary complexes of 3-OST-3 binding to 8-mer 1 (without 6-*O*-sulfation) and 8-mer 3 (with 6-*O*-sulfation) provide a better understanding of the substrate recognition by the enzyme. In superpositions with the ternary complex of 3-OST-1 (Fig. 5C), we demonstrate that the substrate binds along an extended catalytic cleft that is mostly conserved between 3-OST-1 and 3-OST-3. The position of the acceptor glucosamine and conserved catalytic residues used by the two enzymes superimpose very well in the active site. However, the individual uronic acids (saccharides *d* and *f* in 3-OST-3) flanking the acceptor glucosamine in the substrates bound to 3-OST-1 and 3-OST-3 exist in different conformations. For 3-OST-1, the IdoA2S on the reducing side is in the ^1^*C*_4_ conformation while the GlcA on the non-reducing side of the acceptor glucosamine is found in the ^4^*C*_1_ conformation. For 3-OST-3, the IdoA2S saccharides present on both reducing and non-reducing sides of the acceptor display the ^2^*S*_0_ conformation. The ^2^*S*_0_ conformation in the two flanking IdoA2S saccharides alter the trajectories of the oligosaccharide across the substrate binding cleft of 3-OST-3. Two unique residues, *i.e.* Arg173 and Arg190 in the cleft, are in position to form potential hydrogen bonds with the extended substrates, possibly contributing to the distinct substrate binding selectivities between 3-OST-3 and 3-OST-1.

The requirement for 6-*O*-sulfo groups is another distinguishing feature in 3-OST-1 *versus* 3-OST-3 substrate specificity. Previous studies concluded that 3-OST-3 has reduced activity towards saccharide substrates harboring 6-*O*-sulfation, while 3-OST-1 is only active towards 6-*O*-sulfated saccharide substrates.^16^ We reveal here the poor reactivity of 3-OST-3 towards 6-*O*-sulfated oligosaccharides is not due to exclusion of the 6-*O*-sulfation from the enzyme, as originally presumed. The 6-*O*-sulfo groups, in fact, allow for additional interactions with the enzyme which increases the binding affinity between the enzyme and its 6-*O*-sulfated oligosaccharide substrates (Fig. 3A and 6B, respectively). Addition of 6-*O*-sulfo groups inadvertently causes both productive and non-productive bindings between 3-OST-3 and the 8-mer 3 in our structure. It is possible that non-productive binding in this or a similar fashion may contribute to the substantial decrease in the activity to 6-*O*-sulfated oligosaccharide substrates. The discovery of both productive and non-productive binding modes from the crystal structure analysis provides evidence to support this assertion.

Findings from our studies raise an interesting question as to the distinct roles of protein/HS interactions. When a HS chain appropriately interacts with a protein, anticipated biological effects can be observed. It has been widely accepted that specific sulfated saccharide sequences play critical roles in this process.^24^ Our results suggest that overall sulfation levels are also an important contributor in the binding between HS and a protein. HS is present in a complex mixture that contains many different lengths and sulfation patterns. One would expect that a subpopulation of HS may bind to a protein to exhibit the desired biological function, while other subpopulations of HS could bind to the same protein in different orientations to display different biological effects. In the case of 3-OST-3 binding, polarity of substrate binding across the active site may lead to 3-*O*-sulfation, while binding in the opposite direction may result in inhibition. Interestingly, the biological function of fibroblast growth factor 2 (FGF-2) relies on the ability of HS to bind across the same region in FGF-2 monomers, but in different directions. Multi-polarity binding of HS across the same FGF-2 binding site results in a functional dimer that interacts with the FGF receptor for signaling.^25,26^

Structurally guided mutagenesis within the substrate binding pocket of 6-OST and 2-OST have led to greater control of chemoenzymatic synthesis.^27,28^ This study provides clues to regulating 3-OST-3 specificity, generating complex sulfation patterns beyond what is available using wild-type enzyme. The availability of structurally homogeneous HS oligosaccharides with unique sulfation patterns should offer a useful tool to examine the individualized effects of specific sulfated carbohydrate sequences in a biological system.

## Author contributions

RW analyzed the substrate specificity of 3-OST-3 mutants, synthesized 8-mers, measured the binding affinity of 3-OST-3 and 8-mers, and wrote manuscript. AMK conducted the crystal structural trials and prepared different 3-OST mutants. JMK provided technical expertise in refinement and parameterization of oligosaccharides. TP produced the enzymes to support the synthesis of 8-mers. LP and JL designed the study and wrote the manuscript. All authors contributed to manuscript writing and approved the manuscript.

## Conflicts of interest

Y. X. and J. L. are founders of Glycan Therapeutics and have equity. V. P. is an employee of Glycan Therapeutics and has the equity of Glycan Therapeutics. T. P. is an employee of Glycan Therapeutics. Dr Jian Liu's lab at UNC has received a gift from Glycan Therapeutics to support research in glycoscience. R. W., A. M. K., J. M. K. and L. C. P. declare no competing interest.

## Supplementary Material

CB-002-D1CB00079A-s001
