# Supplementary material for: Deciphering the substrate recognition mechanisms of the heparan sulfate 3-O-sulfotransferase-3
Source: RSC Chem Biol. 2021 May 28;2(4):1239–48. doi: 10.1039/d1cb00079a (PMC8341778; doi:10.1039/d1cb00079a)
Supplement: CB-002-D1CB00079A-s001 [file CB-002-D1CB00079A-s001.pdf]

Electronic Supplementary Material (ESI) for RSC Chemical Biology.
This journal is © The Royal Society of Chemistry 2021

Deciphering the substrate recognition mechanisms of the heparan sulfate 3-*O*-sulfotransferase-3

Electronic Supplementary Information

Rylee Wander[1], Andrea M. Kaminski[2], Yongmei Xu[1], Vijayakanth Pagadala[3], Juno M. Krahn[2], Truong Quang Pham[3], Jian Liu[1,*] and Lars C. Pedersen[2]

1. Division of Chemical Biology and Medicinal Chemistry, Eshelman School of Pharmacy, University of North Carolina, Chapel Hill, North Carolina, USA.
2. Genome Integrity and Structural Biology Laboratory, National Institute of Environmental Health Sciences, National Institutes of Health, Research Triangle Park, North Carolina, USA.
3. Glycan Therapeutics, Raleigh, North Carolina, USA.

*Correspondence: jian_liu@unc.edu

This work is supported in part by NIH grants (HL094463, HL144970, GM128484, GM134738, and HL139187) and 1Z1A-ES102645 (LCP) in the Division of Intramural Research program of the National Institute of Environmental Health Sciences, NIH.

Experimental Procedures:

Preparation and expression 3-OST-3 wild type and mutants

The catalytic domain (Gly139-Gly406) of 3-OST-3 was cloned into pGEX4T3 to produce a GST-fusion protein and transformed into BL21(DE3)RIL cells (Stratagene) as previously described [1]. For expression, 20 ml of an overnight culture were added to 12 Fernbach flasks containing 1 L of TB media supplemented with 100 μg/ml ampicillin and 35 μg/ml chloramphenicol. Cells grew at 37 °C with shaking at 275 rpm until OD600 reached 0.65 at which point the temperature was set to 16°C. After 1 hour, IPTG (isopropyl β-D-1-thiogalactopyranose) was added to a final concentration of 400 μM and cells continued to shake overnight at 16°C. Cells were pelleted and resuspended in 120 ml of sonication buffer consisting of 20 mM sodium/potassium phosphate pH 7.2 and 625 mM NaCl and supplemented with Protease Complete EDTA-free (Roche) protease inhibitors. Cells were sonicated and the cell debris pelleted by centrifugation. The soluble fraction was loaded on to Glutathinone-Sepharose 4B (GE healthcare) resin in-batch and washed repeatedly with sonication buffer. 3-OST-3 was cleaved from the bound GST with 600 units of thrombin (Sigma) in a 50 ml conical tube on a shaker at 4 °C overnight. Protein was concentrated and further purified on a Superdex 200 26/60 (GE healthcare) column equilibrated in 25 mM Tris pH 7.5 and 500 mM NaCl. Fractions containing 3-OST-3 were pooled and PAP was added to 100 μM. The NaCl concentration was reduced to 125 mM by addition of 25 mM Tris pH 7.5 and 100 μM PAP. Protein was concentrated to ~ 3 mg/ml, supplemented with PAP to a final concentration of 4 mM, then further concentrated to ~15 mg/ml. For protein used in biochemical studies, the binding to Gluthathione-4B was performed on a column and a Superdex 75 16/60 (GE Healthcare) was used for gel filtration. For mutagenesis studies, protein was expressed in the pGEXM vector [2] following the same protocol but cleaved with TEV (Tobacco Etch Virus) protease rather than thrombin.

Protein Co-crystallization

Crystals of the 3-OST-3/PAP/8-mer 1 complex were obtained using sitting drop vapor diffusion at 20°C by mixing 325 nl of 3-OST-3 at 14 mg/ml in 25 mM Tris pH 7.5, 125 mM NaCl, 4 mM PAP, and 5 mM 8-mer 1 with 200 nl of reservoir solution consisting of 200 mM potassium iodide and 20% (w/v) polyethylene glycol (PEG) 3350. For data collection, 1 μl of cryo solution consisting of 15% ethylene glycol, 85% reservoir, and 5mM 8-mer 1 was added directly to the drop, followed by transfer of the crystal to 100% cryo solution. The crystal was then flash frozen in liquid nitrogen.

Crystals of the 3-OST-3/PAP/8-mer 3 with 6-*O*-sulfo groups were obtained using sitting drop vapor diffusion at 20°C by mixing 250 nl of 15.5 mg/ml 3-OST-3 in 25 mM Tris pH 7.5, 125 mM NaCl, 4 mM PAP, and 5 mM 8-mer 3 with 250 nl of 100 mM bicine pH 9.0 and 10% (v/v) 2-methyl-2,4-pentanediol (MPD). For data collection, 1 μl of cryo solution consisting of 26% ethylene glycol and 74% reservoir was added to the drop, followed by the addition of another 1 μl. The crystal was then flash frozen in liquid nitrogen.

All data were collected on a Rigaku MicroMax 007HF X-ray generator using a Dectris P200 detector at -173°C and processed with HKL3000. The structures were both solved independently in Phenix[3]. The protein component of PDB ID code 1T8U [1] was used as a search model with Phaser[4]. All refinement was carried out with multiple cycles of refinement in Phenix and manual model building in Coot [5,6] (Table 1).

Chemoenzymatic Synthesis of 8-mer 1, 8-mer 2, 8-mer 3 and 8-mer 4

The chemoenzymatic method of synthesizing oligosaccharides as outlined in Xu *et al*. and Zhang *et al*. was utilized for the synthesis of 8-mer 1, 8-mer 2, 8-mer 3, and 8-mer 4 [7,8]. 3-*O*-sulfations were installed using 3-OST-3, as shown in Wang *et al*. [9]. Synthesis was initiated through elongation of a monomer of *para*-nitrophenyl glucuronide (GlcA-pNP, purchased from Carbosynth), using the enzyme pmHS2 and uridine-diphosphate sugar nucleotide donors, UDP-GlcNTFA (*N*-trifluoroacetylated glucosamine) and

UDP-GlcA. Both GlcNTFA and UDP-GlcA were made in-house [10]. Briefly, GlcA-pNP (3.2mM) was added to a solution containing 25 mM Tris pH 7.2, 5 mM $MnCl_2$, 60 µg/ml pmHS2, 4.5 mM UDP-GlcNTFA and incubated at 37°C overnight. Product formation was tracked by analysis on HPLC using a polyamine column (PAMN, from Waters), monitoring absorbance at 310 nm, and product identity was verified by electrospray ionization mass spectrometry (ESI-MS). Next, the disaccharide (3.2 mM) was added to a solution containing 25 mM Tris pH 7.2, 5 mM $MnCl_2$, 60 µg/ml pmHS2, 4.5 mM UDP-GlcA and incubated at 37°C overnight. Again, product formation and identity were confirmed using HPLC and ESI-MS, respectively. These verification techniques were used after each step of synthesis moving forward. These elongation steps were repeated until a 6-mer structure was obtained. The product of each step was purified using a C_{18} column (0.75 x 20 cm; Biotage) and eluted on a linear gradient from 1-100% acetonitrile in H_2O and 0.1% trifluoroacetic acid (TFA) in 60 minutes with a flow rate of 4 ml/min. Once the chain reached 6 saccharides, the GlcNTFA saccharides were detrifluroacetylated by addition of 0.1 M LiOH and maintenance of pH above 12 at room temperature for 10 min. The formation of the detrifluoroacetylated product was monitored by ESI-MS. The oligosaccharide glucosamine saccharides were then *N*-sulfated using *N*-sulfotransferase (C-terminal domain of *N*-deacetylase/*N*-sulfotransferase 1). The compound was added to a solution containing 50 mM MOPS pH 7, 32 µg/ml *N*-sulfotransferase, and PAPS (1.5 molar equivalent of free amino groups) and incubated at 37°C overnight. Both PAPS and *N*-sulfotransferase were prepared in-house [10]. Product was purified by ion exchange chromatography on a Q-Sepharose column (GE Healthcare). Next, epimerization and 2-*O*-sulfation were carried out in a combined reaction to convert GlcA (saccharide *f*, Fig. 1B) to 2-*O*-sulfated iduronic acid. The oligosaccharide was added to a solution containing 50 mM MOPS pH 7.0, 2 mM $MnCl_{2,}$ PAPS (1.5 x molar excess), C_5 epimerase enzyme (5 µg/mL), and 2-*O*-sulfotransferase (15 µg/mL) and the mixture was incubated at 37°C overnight. Product was again purified using a Q-Sepharose column. The chain was then further elongated by two more saccharides, this time utilizing a UDP-GlcNAc monomer for the final

elongation step, rather than UDP-GlcNTFA (8 saccharides total). The epimerization/2-*O*-sulfation step was next carried out again to convert saccharide *d*. The resultant oligosaccharide was 8-mer 1. At this point, octasaccharides were split into groups to obtain the remaining three structures used (8-mers 2, 3 and 4). 8-mer 3 was obtained by reacting the 8-mer 1 structure with 6-*O*-sulfotransferase until 6-*O*-sulfation of all four glucosamines was achieved. Briefly, the 8-mer 1 structure (0.5 mM) was added to buffer containing 50 mM MOPS (pH 7.0, 6-OST-1 (50 µg/mL), 6-OST-3 (50 µg/mL), and PAPS (1.5 molar equivalent of 6-hydroxyl groups) and incubated overnight at 37°C. Purification via a Q-Sepharose column resulted in 8-mer 3. 8-mer 2 was obtained by reacting 8-mer 1 with 3-OST-3 to install the 3-*O*-sulfation on glucosamine e. 8-mer 1 was added to a solution containing 50 mM MOPS pH 7.0), 5 mM $MnCl_2$, 5 mM $MgCl_{2,}$ PAPS (1.5 x molar equivalent), and 3-OST-3 (15 µg/mL) and incubated at 37°C overnight. Purification by a Q-Sepharose column and verification of product identity by ESI-MS yielded 8-mer 2. This same 3-OST-3 step was taken for 8-mer 4, which was then further modified through 6-*O*-sulfation as described for 8-mer 3 and purified a final time by a Q-Sepharose column.

The final structures of four octasaccharides were confirmed by electrospray ionization mass spectrometry (ESI-MS). The *m/z* values observed in the analysis for each octasaccharides were as follows: 8-mer 1 MW: 1929, expected *m/z* values for quadruply and triply charged ions are: 481.3 and 642.0, observed peaks: 481.8 and 642.4 (Suppl. Fig. S1); 8-mer 2 MW: 2048, expected *m/z* values for quintuply and quadruply charged ions: 408.6 and 511.0, observed peaks: 408.9 and 511.1 (Suppl. Fig. S2); 8-mer 3 MW: 2249, expected *m/z values* for sextuple and quintuply charged ions: 373.8 and 448.8, observed peaks: 374.3 and 449.2 (Suppl. Fig S3); 8-mer 4 MW: 2368, expected *m/z values* for sextuply and quintuply charged ions: 393.7 and 472.6, observed peaks: 393.8 and 472.7 (Supple. Fig. S4). Oligosaccharide purity was determined by HPLC analysis with a ProPac A1 column. All synthesized oligosaccharides were >93% pure.

Enzyme Titration Assay

Reactions containing either substrate 8-mer 1 (5 μM) or substrate 8-mer 3 (5 μM), [^{35}S]PAPS (10^6 cpm and 20 μM), 50 mM MOPS, 5 mM $MgSO_4$, 5 mM $MnCl_2$, and varying concentrations of 3-OST-3 (0-5.3 μM) were incubated at 37°C for 1 hour. Reactions were then quenched by the addition of 900 μl UPAS (50 mM NaOAc, pH 5.5, 150 mM NaCl, 6 M urea, 1 mM EDTA, and 0.01% Triton X-100) and purified on a 150 μL DEAE-Sepharose (Sigma) column. The ^{35}S-labeled products were eluted in 1 mL of 1 M NaCl, mixed with 3 mL Econo-Safe scintillation cocktail (Research Products International), and counted via a scintillation counter.

HPLC Analysis

Reactions were analyzed by a ProPac A1 column using buffer A (20 mM NaOAc, pH 5.0) and buffer B (20 mM NaOAc, 2M NaCl, pH 5.0). A linear gradient from 35-100% buffer B over 60 minutes with a flow rate of 1 ml/min, was used for elution, with monitoring at 310 nm (corresponds to the absorbance of the *para*-nitrophenol on the reducing end of the oligosaccharides).

Determination of the inhibition of 3-OST-3 by different 8-mers

Reactions containing substrate 8-mer 1 (10 μM), 3-OST-3 (19 nM or 0.62 μg/mL, ~32kDa), [^{35}S]PAPS (10^6 cpm and 4 μM), 50 mM MOPS, 5 mM $MgSO_4$, 5 mM $MnCl_2$, and varying concentrations of inhibitor oligosaccharides (0-5 μM) were incubated at 37°C for 1 hour. Reactions were then quenched by the addition of 900 μl UPAS (50mM NaOAc, pH 5.5, 150 mM NaCl, 6 M urea, 1 mM EDTA, and 0.01% Triton X-100) and purified on a 150 μL DEAE-Sepharose (Sigma) column. The ^{35}S-labeled 8-mers were eluted in 1

mL of 1 M NaCl, mixed with 3 mL Econo-Safe scintillation cocktail (Research Products International), and counted via a scintillation counter.

Determination of the binding affinity between 3-OST-3 and 8-mers using isothermal titration calorimetry (ITC)

His-tagged 3-OST-3 was expressed in *E. coli* and purified by Ni-Sepharose column and heparin-Sepharose column [1]. The protein (0.3 mg/mL) was thawed from -80°C, and PAP was added at a 3 × molar excess over the estimated final protein concentration. Protein was then concentrated using a 10 kDa Amicon Ultra-15 centrifugal filter (Millipore Sigma) pre-rinsed with the ITC buffer (100 mM Phosphate, 100 mM NaCl, PAP 3X desired protein concentration, pH 7.0) to remove any residual glycerol. The protein was concentrated to the desired concentration (~1.0 mg/mL) then buffer-exchanged 7 times with ITC buffer. Final protein concentration was determined by Bradford assay using Coomassie Plus – The Better Bradford Assay Reagent (Thermo Scientific). In each experiment various concentrations of bovine serum albumin were used to develop a standard curve. The oligosaccharide analytes were diluted to desired concentration using ITC buffer from stocks prior to the analysis. The ITC analysis was carried out at UNC Macromolecular Interactions Facility using a Micro-Cal Auto ITC-200.

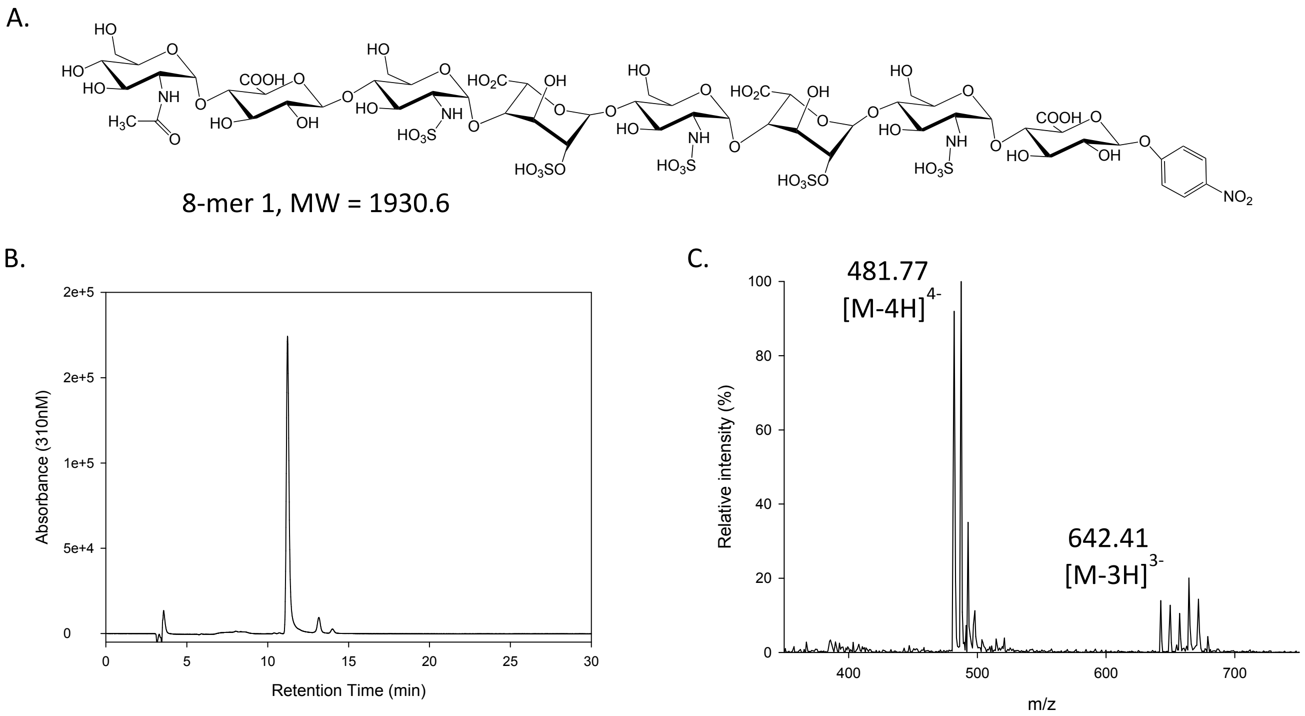

Suppl. Fig. S1. **HPLC and ESI-MS analysis of 8-mer 1.** A) 8-mer 1 structure B) Anion exchange HPLC chromatogram of 8-mer 1. C) ESI-MS spectrum of 8-mer 1. The compound was observed in its quadruply and triply charged states. The measured molecular weight of 8-mer 1 was 1930.2, which is very close to the calculated value (1930.6). Additional peaks are the result of sodium adducts.

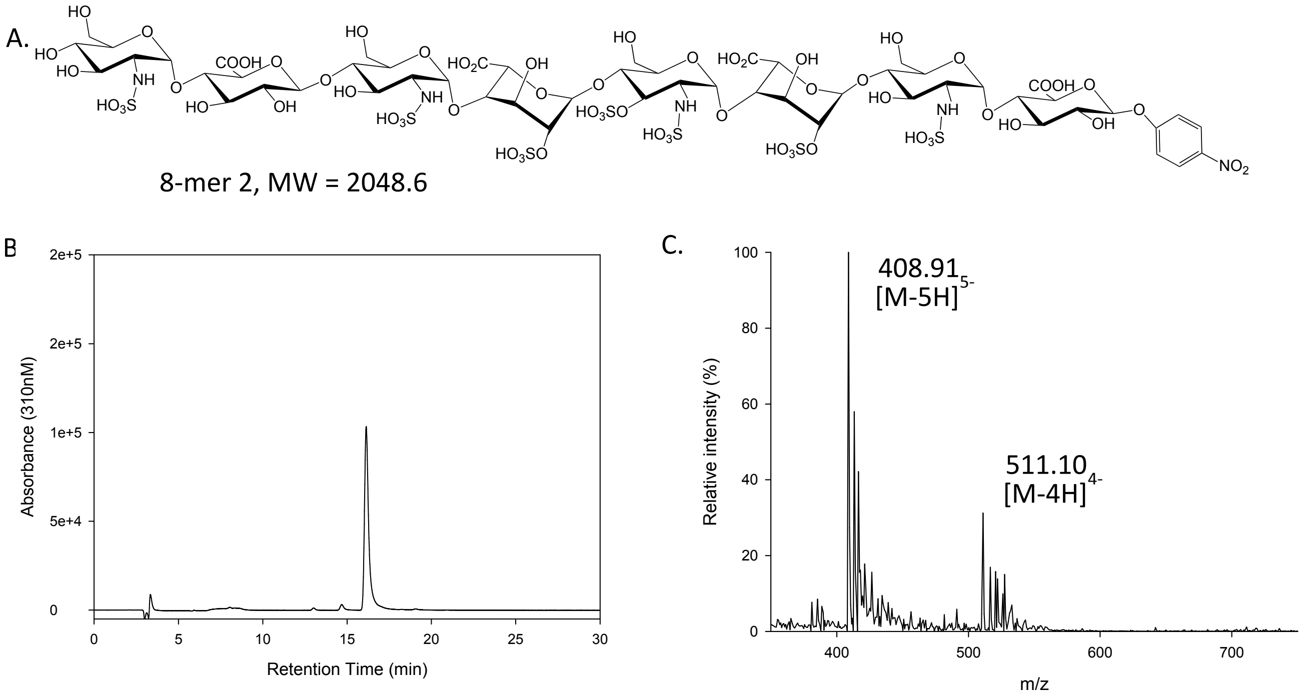

Suppl Fig. S2. **HPLC and ESI-MS analysis of 8-mer 2.** A) 8-mer 2 structure B) Anion exchange HPLC chromatogram of 8-mer 2. C) ESI-MS spectrum of 8-mer 2. The compound was observed in its quintuply and quadruply charged states. The measured molecular weight of 8-mer 2 was 2048.4, which is very close to the calculated value (2048.6). Additional peaks are the result of sodium adducts.

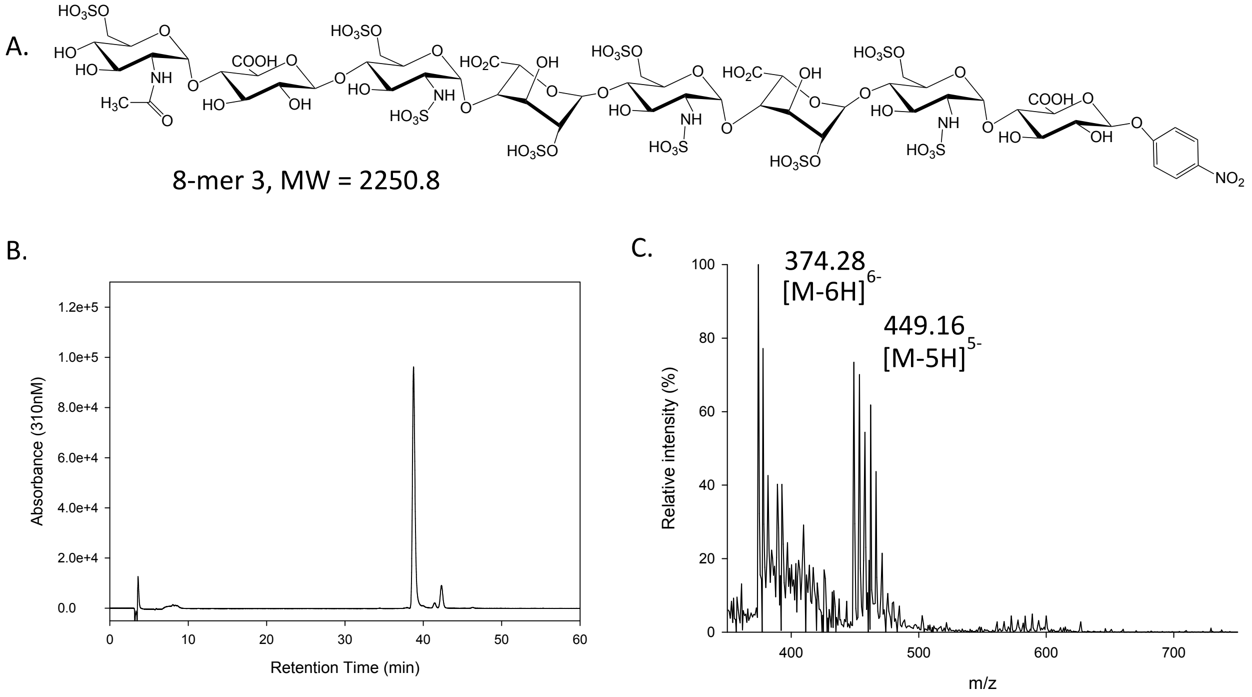

Suppl. Fig. S3. **HPLC and ESI-MS analysis of 8-mer 3**. A) 8-mer 3 structure B) Anion exchange HPLC chromatogram of 8-mer 3. C) ESI-MS spectrum of 8-mer 3. The compound was observed in its sextuply and quintuply charged states. The measured molecular weight of 8-mer 3 was 2251.0, which is very close to the calculated value (2250.8). Additional peaks are the result of sodium adducts.

A.

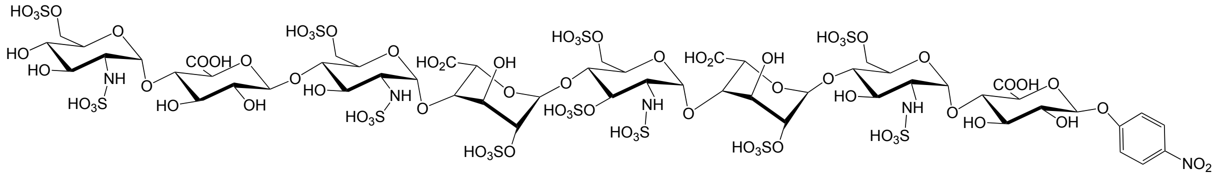

8-mer 4, MW = 2368.9

B.

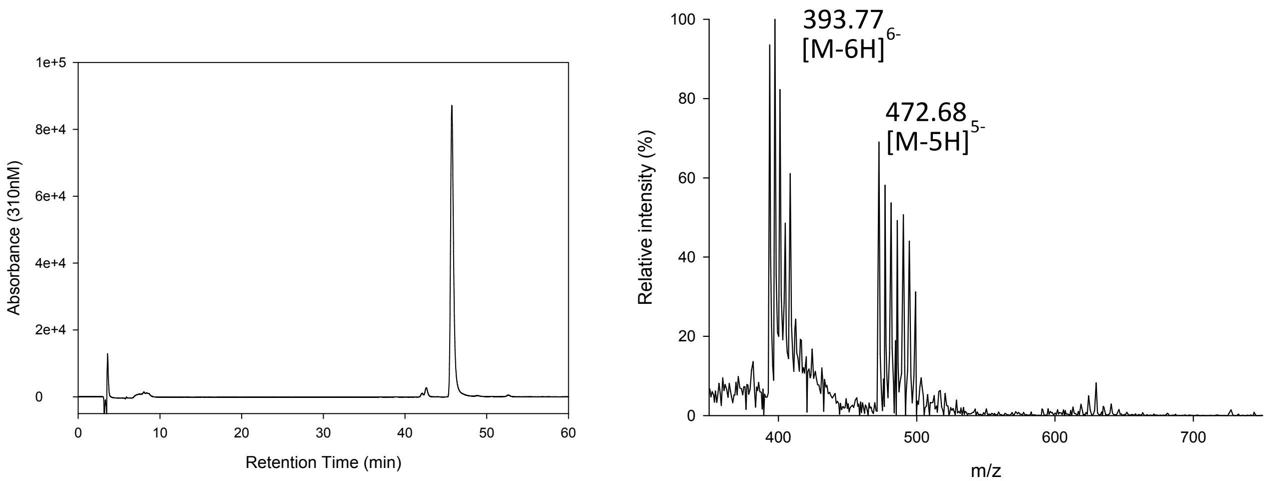

Suppl. Fig. S4. **HPLC and ESI-MS analysis of 8-mer 4**. A) 8-mer 4 structure B) Anion exchange HPLC chromatogram of 8-mer 4. C) ESI-MS spectrum of 8-mer 4. The compound was observed in its sextuply and quintuply charged states. The measured molecular weight of 8-mer 4 was 2368.8, which is very close to the calculated value (2368.9). Additional peaks are the result of sodium adducts.

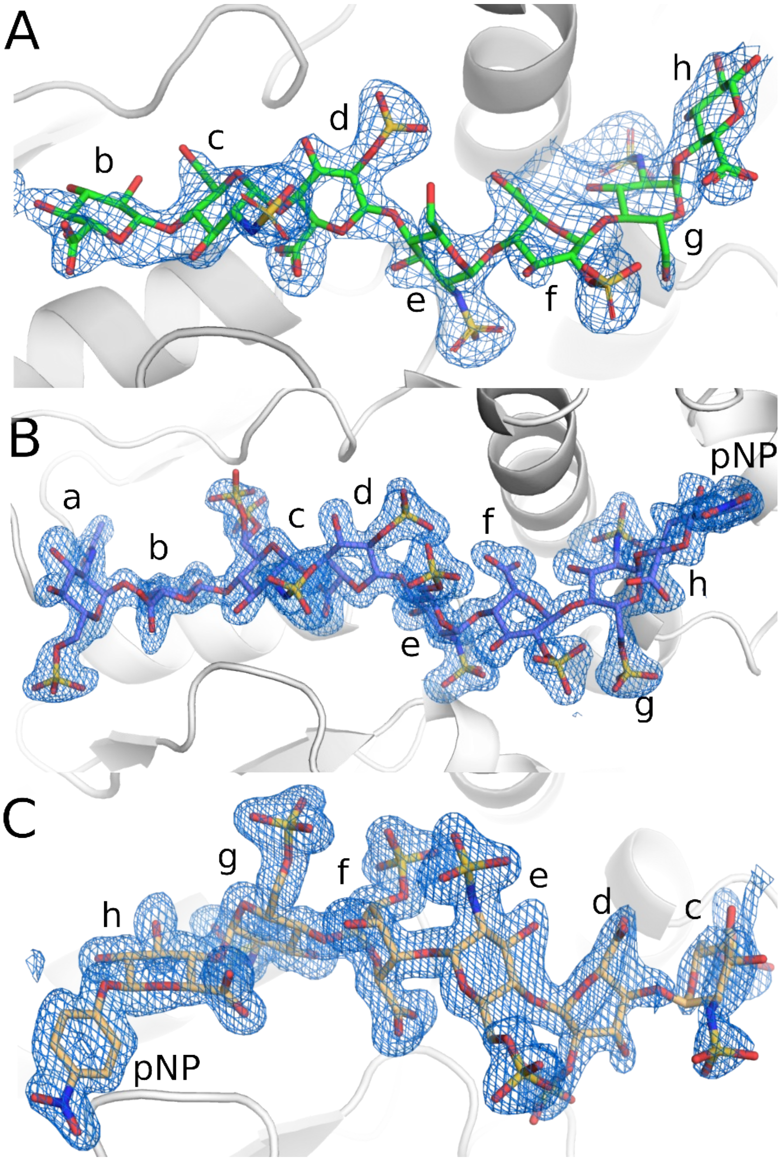

Suppl. Fig. S5. Electron density of octasaccharides binding to 3-OST-3

Displayed are *Fo-Fc* simulated annealing omit maps (blue) of (A) 8-mer 1 binding to 3OST-3 (B) 8-mer 3 binding in productive mode to 3OST-3 and (C) 8-mer 3 binding in nonproductive mode to 3-OST-3. Octasaccharides colored as in Figures 5 and 6. Map in panel A is contoured at 2.5σ, while maps in B and C are contoured at 3.0σ. Saccharides with electron density in the respective binding pockets are labeled a-h with the para-nitrophenol labeled pNP

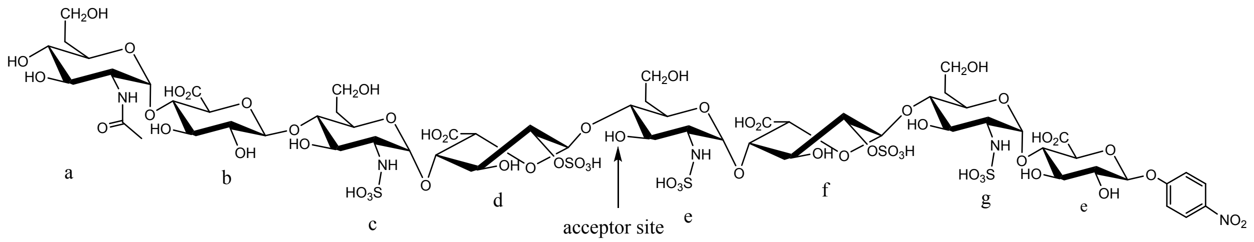

Productive binding orientation

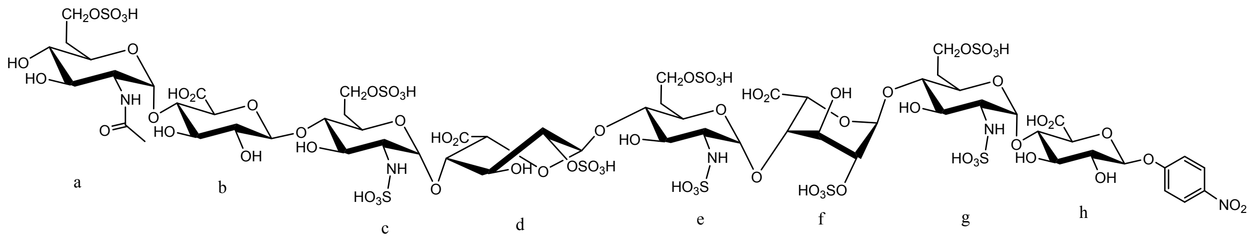

Nonproductive binding orientation

Suppl. Fig. S6. Different conformations of saccharide *f* of 8-mer 3 in productive and nonproductive orientation when the octasaccharide interacts with 3-OST-3.

```
h3OST3a   139 GTLALLLDEGSKQLPQAIIIGVKKGGTRALLEFLRVHPDVRAVGAEPHFFD  189
m3OST1     54 ---------STQQLPQTIIIGVRKGGTRALLEMLSLHPDVAAAENEVHFFD   95
                       .::****:*****:*********:* :**** *.  * ****

h3OST3a   190 R--SYDKGLAWYRDLMPRTLDGQITMEKTPSYFVTREAPARISAMSKDTKL  238
m3OST1     96 WEEHYSQGLGWYLTQMPFSSPHQLTVEKTPAYFTSPKVPERIHSMNPTIRL  146
                  *.:**.**   ** :   *:*:****:**.: :.* ** :*.   :*

h3OST3a   239 IVVVRDPVTRAISDYTQTLSKRP----DIPTFESLTFKNRTAGLIDTSWSA  285
m3OST1    147 LLILRDPSERVLSDYTQVLYNHLQKHKPYPPIEDLLMRD---GRLNLDYKA  194
              ::::***  *.:*****.*  :      .*.:*.* :::   * :: .:.*

h3OST3a   286 IQIGIYAKHLEHWLRHFPIRQMLFVSGERLISDPAGELGRVQDFLGLKRII  336
m3OST1    195 LNRSLYHAHMLNWLRFFPLGHIHIVDGDRLIRDPFPEIQKVERFLKLSPQI  245
              :: .:*  *: :***.**: :: :*.*:*** **  *: :*: ** *.  *

h3OST3a   337 TDKHFYFNKTKGFPCLKKAEGSSRPHCLGKTKGRTHPEIDREVVRRLREFY  387
m3OST1    246 NASNFYFNKTKGFYCLRD---SGKDRCLHESKGRAHPQVDPKLLDKLHEYF  293
              . .:********* **:.   *.: :** ::***:**::* ::: :*:*::

h3OST3a   388 RPFNLKFYQMTGHDFGWDG  406
m3OST1    294 HEPNKKFFKLVGRTFDWH-  311
              :  * **:::.*: *.*.
```

Suppl. Fig. S7. Sequence alignment of human 3-OST-3a to mouse 3-OST-1

Sequence alignment of human 3-OST-3a versus mouse 3-OST-1 catalytic domains based on crystal structures. Residues highlighted in red are the catalytic base glutamates (Glu184 for 3-OST-3 and Glu90 for 3-OST-1) and the catalytic acids (Lys162 for 3-OST-3 and Lys68 for 3-OST-1). Conserved residues lining the heparan sulfate binding cleft that greatly affect catalytic activity for 3-OST-3 when mutated are colored magenta [1]. Residues that line the binding cleft that are not conserved and were mutated in this study are colored green.

Supplementary Table S1. 8-mer 1 hydrogen bonding interactions with 3-OST-3 mol B

3-OST-3	8-mer 1(position); conformation	Distance (Å)
	**GlcA(*b*); $^4C_1$**	
H362 NE2	O3	2.5
E170 OE2	O3	3.3
R173 NH2	O6B (CO_2)	2.73
	**GlcNAc(*c*); $^4C_1$**	
	None	
	**IdoA2S(*d*); $^2S_0$**	
R166 NE	O61 (CO_2)	2.8
R166 NH2	O62 (CO_2)	3.0
K215 NZ	O61 (CO_2)	3.1
K259 NZ	O1S (2S)	2.9
T367 OG	O3	3.1
K368 NZ	O61 (CO_2)	3.0
R370 NH2	O3S (2S)	3.0
	**GlcNS (*e*); $^4C_1$**	
E184 OE2	O3 *	2.5
S218 N	O35 (NS)	3.2
S218 OG1	O35 (NS)	3.3
	**IdoA2S (*f*); $^2S_0$**	
K161 NZ	O5	2.7
K161 NZ	O62 (CO_2)	2.8
Q255 NE2	O61 (CO_2)	2.8
K259 NZ	O61 (CO_2)	2.6
	**GlcNS (*g*); $^4C_1$**	
T256 OG1	O2S (NS)	3.2
T256 OG1	O3	3.2
W283 NE1	O1S (NS)	2.7
	**Sodium ion**	
**Protein**	**8mer 1**	**Distance (Å)**
D252 OD1		2.4
D252 O		2.6
T256 OG1		2.4
	GlcNS (g) O3	2.6
	GlcNS (g) O2S (NS)	2.6
	IdoAS (f) O62 (CO_2)	3.0

Supplementary Table S2.

8mer 3 potential H-bond interactions with 3OST-3 mol B (Productive Binding)		
3OST-3 (lattice mol)	8mer 3(position); conformation	Distance (Å)
	**GlcNAc6S (a); $^4C_1$**	
R173 NH2	O5	3.2
R173 NH2	O9 (6S)	3.2
G357 N	O (Ac)	2.8
Q396 OE1 (A)	O4	2.7
Q396 NE2 (A)	O3	2.9
	**GlcA(b); $^4C_1$**	
E170 OE1	O3	3.1
E170 OE2	O3	3.0
E170 OE2	O2	2.5
	**GlcNS6S (c); $^4C_1$**	
G365 N	O4S conf A; O5S conf B (6S)	3.0; 2.8
	**IdoA2S(d); $^2S_0$**	
R166 NE	O61 (CO_2)	2.9
R166 NH2	O62 (CO_2)	2.9
K215 NZ	O61 (CO_2)	3.2
K215 NZ	O62 (CO_2)	3.3
K259 NZ	O2S (2S)	2.9
T367 OG	O3	3.0
K368 NZ	O61 (CO_2)	2.9
R370 NH2	O3S (2S)	2.9
	**GlcNS6S (*e*); $^4C_1$**	
E184 OE2	O3 *	2.6
S218 N	O2S (NS)	3.1
Q255 NE2	O4	3.3
K259 NZ	O5S (6S)	3.1
	**IdoA2S (*f*); $^2S_0$**	
K161 NZ	O5	2.9
K161 NZ	O62 (CO_2)	2.9
R190 NH2	O3	2.8
Q255 NE2	O61 (CO_2)	2.8
K259 NZ	O61 (CO_2)	2.8
	**GlcNS6S (*g*); $^4C_1$**	
K161 NZ	O3	3.3
T256 OG1	O3	3.0
T256 OG1	N	2.9
W283 NE1	O2S	2.74
Sodium ion		
Protein	8mer 1	Distance (Å)

D252 OD1		2.3
D252 O		2.4
T256 OG1		2.4
	GlcNS6S (*g*) O3	2.4
	GlcNS6S (*g*) O3S (NS)	2.6
	IdoA2S (*f*) O62 (CO_2)	2.7

Supplementary Table S2. 8-mer 3 potential H-bond interactions with 3OST-3 mol B (Productive Binding)

3-OST-3 (lattice mol)	8-mer 3(position); conformation	Distance (Å)
	**GlcNAc6S (a); $^4C_1$**	
R173 NH2	O5	3.2
R173 NH2	O9 (6S)	3.2
G357 N	O (Ac)	2.8
Q396 OE1 (A)	O4	2.7
Q396 NE2 (A)	O3	2.9
	**GlcA(b); $^4C_1$**	
E170 OE1	O3	3.1
E170 OE2	O3	3.0
E170 OE2	O2	2.5
	**GlcNS6S (c); $^4C_1$**	
G365 N	O4S conf A; O5S conf B (6S)	3.0; 2.8
	**IdoA2S(d); $^2S_0$**	
R166 NE	O61 (CO_2)	2.9
R166 NH2	O62 (CO_2)	2.9
K215 NZ	O61 (CO_2)	3.2
K215 NZ	O62 (CO_2)	3.3
K259 NZ	O2S (2S)	2.9
T367 OG	O3	3.0
K368 NZ	O61 (CO_2)	2.9
R370 NH2	O3S (2S)	2.9
	**GlcNS6S (*e*); $^4C_1$**	
E184 OE2	O3 *	2.6
S218 N	O2S (NS)	3.1
Q255 NE2	O4	3.3
K259 NZ	O5S (6S)	3.1
	**IdoA2S (*f*); $^2S_0$**	
K161 NZ	O5	2.9
K161 NZ	O62 (CO_2)	2.9
R190 NH2	O3	2.8
Q255 NE2	O61 (CO_2)	2.8
K259 NZ	O61 (CO_2)	2.8
	**GlcNS6S (*g*); $^4C_1$**	
K161 NZ	O3	3.3
T256 OG1	O3	3.0
T256 OG1	N	2.9
W283 NE1	O2S	2.74
**Sodium ion**		
**Protein**	**8mer 1**	**Distance (Å)**
D252 OD1		2.3
D252 O		2.4
T256 OG1		2.4

	GlcNS6S (*g*) O3	2.4
	GlcNS6S (*g*) O3S (NS)	2.6
	IdoA2S (*f*) O62 (CO_2)	2.7

Supplementary Table S3. 8-mer 3 H-bond interactions with 3OST-3 mol A (Nonproductive Binding)

3-OST-3 (lattice mol)	8-mer 3 (position); conformation	Distance (Å)
	**GlcNS6S (*c*); $^4C_1$**	
S284 N	O5S (6S)	3.2
R334 conf B NH1 (B)	O2S	2.9
R334 conf B NH2	O3	3.0
	**IdoA2S(*d*); $^2S_0$**	
R190 NE	O2S (2S)	2.5
R190 NH2	O3S (2S)	3.0
	**GlcNS6S (*e*); $^4C_1$**	
K259 NZ	O1S (NS)	3.0
R260 NH2	O3S (NS)	2.7
K333 NZ (B)	O6S (6S)	
	**IdoA2S (*f*); $^1C_4$**	
K259 NZ	O3S (2S)	2.8
R190 NH1	O61 (CO2)	2.8
R190 NH2	O62 (CO2)	2.7
Q255 NE2	O1S (2S)	3.3
Q255 NE2	O2S (2S)	3.2
R334 NH2 (B)	O3	3.3
	**GlcNS6S (*g*); $^4C_1$**	
R166 NH1	O2S (NS)	2.9
E184 OE1	O3	2.8
R215 NZ	O1S (NS)	2.9
R215 NZ	O3S (NS)	2.9
K259 NZ	O5S (6S)	3.0
	**GlcA (*h*) ; $^4C_1$**	
R166 NH1	O3	3.0

Reference

1 Moon, A.; Edavettal, S. C.; Krahn, J. X.; Munoz, E. M.; Negishi, M.; Linhardt, R. J.; Liu, J.; Pedersen, L. C. *J. Biol. Chem.*, 2004, **279**, 45185.

2 Moon, A. F.; Pryor, J. M.; Ramsden, D. A.; Kunkel, T. A.; Bebenek, K.; Pedersen, L. C. *Nat. Struct. Mol. Biol.* , 2014, **21**, 253.

3 Adams, P. D.; Afonine, P. V.; Bunkóczi, G.; Chen, V. B.; Davis, I. W.; Echols, N.; Headd, J. J.; Hung, L. W.; Kapral, G. J.; Grosse-Kunstleve, R. W.; McCoy, A. J.; Moriarty, N. W.; Oeffner, R.; Read, R. J.; Richardson, D. C.; Richardson, J. S.; Terwilliger, T. C.; Zwart, P. H. *Acta Crystallogr. D Biol. Crystallogr.*, 2010, **66**, 213.

4 McCoy, A. J. *J. Appl. Crystallogr.*, 2007, **40**, 658.

5 Emsley, P.; Cowtan, K. *Acta Crystallogr D. Biol Crystallogr*, 2004, **60(pt 12)**, 2126.

6 Emsley, P.; Lohkamp, B.; Scott, W. G.; Cowtan, K. *Acta Crytallogr. D Biol. Crystallogr.*, 2010, **66**, 486.

7 Xu, Y.; Cai, C.; Chandarajoti, K.; Hsieh, P.; Lin, Y.; Pham, T. Q.; Sparkenbaugh, E. M.; Sheng, J.; Key, N. S.; Pawlinski, R. L.; Harris, E. N.; Linhardt , R. J.; Liu, J. *Nat Chem Biol*, 2014, **10**, 248.

8 Zhang, X.; Pagadala, V.; Jester, H. M.; Lim, A. M.; Pham, T. Q.; Liu, J.; Linhardt, R. J. *Chem. Sci.*, 2017, **8**, 7932.

9 Wang, Z.; Hsieh, P.-H.; Xu, Y.; Thieker, D.; Chai, E. J. E.; Xie, S.; Cooley, B.; Woods, R. J.; Chi, L.; Liu, J. *J. Am. Chem. Soc.*, 2017, **139**, 5249.

10 Xu, Y.; Chandarajoti, K.; Zhang, X.; Pagadala, V.; Dou, W.; Hoppensteadt, D. M.; Sparkenbaugh, E.; Cooley, B.; Daily, S.; Key, N. S.; Severynse-Stevens, D.; Fareed, J.; Linhardt, R. J.; pawlinski, R.; Liu, J. *Sci. Transl. Med.*, 2017, **9**, eaan5954.
